# Toward Smart Footwear to Track Frailty Phenotypes—Using Propulsion Performance to Determine Frailty

**DOI:** 10.3390/s18061763

**Published:** 2018-06-01

**Authors:** Hadi Rahemi, Hung Nguyen, Hyoki Lee, Bijan Najafi

**Affiliations:** 1 Interdisciplinary Consortium on Advanced Motion Performance (iCAMP), Michael E. DeBakey Department of Surgery, Baylor College of Medicine, Houston, TX 77030, USA; rahemi@circulationconcepts.com (H.R.); hung.nguyen@bcm.edu (H.N.); Lee.hyoki@gmail.com (H.L.); 2 Circulation Concepts Inc., Houston, TX 77030, USA; 3 BioSensics LLC, Watertown, MA 02472, USA

**Keywords:** frailty, gait, artificial neural network, propulsion, aging, wearable sensor, walking, smart footwear

## Abstract

Frailty assessment is dependent on the availability of trained personnel and it is currently limited to clinic and supervised setting. The growing aging population has made it necessary to find phenotypes of frailty that can be measured in an unsupervised setting for translational application in continuous, remote, and in-place monitoring during daily living activity, such as walking. We analyzed gait performance of 161 older adults using a shin-worn inertial sensor to investigate the feasibility of developing a foot-worn sensor to assess frailty. Sensor-derived gait parameters were extracted and modeled to distinguish different frailty stages, including non-frail, pre-frail, and frail, as determined by Fried Criteria. An artificial neural network model was implemented to evaluate the accuracy of an algorithm using a proposed set of gait parameters in predicting frailty stages. Changes in discriminating power was compared between sensor data extracted from the left and right shin sensor. The aim was to investigate the feasibility of developing a foot-worn sensor to assess frailty. The results yielded a highly accurate model in predicting frailty stages, irrespective of sensor location. The independent predictors of frailty stages were propulsion duration and acceleration, heel-off and toe-off speed, mid stance and mid swing speed, and speed norm. The proposed model enables discriminating different frailty stages with area under curve ranging between 83.2–95.8%. Furthermore, results from the neural network suggest the potential of developing a single-shin worn sensor that would be ideal for unsupervised application and footwear integration for continuous monitoring during walking.

## 1. Introduction

By 2060, it is predicted that 1 in 4 Americans will be over 65 or older [1]. The surge of the aging population could be medically translated into an increase in geriatric condition and syndromes, such as frailty. Frailty syndrome is the loss in physiological reserve of a person and it is highly prevalent in older population. It has been shown to be an indicator of increased fall risk in older adults [2,3,4], a predictor of adverse outcomes of medical intervention [5,6,7,8], and it is associated with reduced quality of life [9]. Furthermore, frailty may result in an increase in healthcare cost with higher readmission cost and more specialist and emergency visits [10].

In recent years, multiple tools have been developed to capture age-related markers that might be indicators of decreased physiological reserve and diminished resistance to stressors [11,12]. For example, Fried et al. [13] initially hypothesized five frailty phenotypes, including shrinking, weakness, slowness, exhaustion, and low activity. Using these phenotypes, they then categorized an individual as either non-frail (zero phenotype is present), pre-frail (one or two phenotypes are present), or frail (three or more phenotypes are present). These phenotypes were then validated through a Cardiovascular Health Study [13], with cohorts of over five thousand participants that were followed over a seven-year period. This frailty classification was developed and recognized as “Fried Criteria” or “Frailty Phenotypes” and has been widely used to assess frailty in clinical setting. However, one of the drawbacks of the Fried Criteria is that some of the criteria are based on self-report (e.g., exhaustion, low activity, and shrinking); therefore, it is semi-objective and it requires trained personnel to conduct the assessment, especially when evaluating patients with cognitive impairment. Furthermore, since assessment using Fried Criteria provides categorical values to describe frailty, its sensitivity to ascertain the effectiveness of any intervention (e.g., nutrition, exercise, etc.), side effects of an intervention (e.g., medication, frailty induced by offloading boot, etc.), or frailty trajectories over time is limited [14,15].

Recent studies have suggested that gait speed is the strongest indicator to predict adverse outcomes, such as mobility disability, falls, or hospitalization, as described in a systematic review by Schwenk et al. [16]. Despite this fact, little efforts have been done to extract other gait related parameters that could describe different frailty phenotypes. Recently, there has been an increase in the utility of using wearable inertial sensors, which are often embedded with accelerometer and gyroscope, to measure frailty stages [17,18,19,20,21,22,23,24,25,26,27,28] with the potential implications of reducing burden on the medical staff, objectively assessing frailty, and monitoring changes in frailty over time. These studies attempted to quantify key physical frailty phenotypes, as described by Fried et al. (e.g., slowness, weakness, exhaustion, low activity, and shrinking), using wearable technologies and incorporating different functional performance test scenarios. These wearable technologies have the potential to remotely screen and/or track changes in frailty stages, including facilitating in-place and remote assessment, which can provide continuous and unsupervised monitoring for patients who have limited access to the clinics for assessment and follow ups. The development of simplified and innovative gait-related metrics that are sensitive to identifying frailty phenotypes can help to promote the integration of sensors in smart footwear devices (e.g., smart socks, smart brace, smart insoles, smart shoes, smart offloading boots, etc.) to help assess frailty during daily living activity such as walking. Currently, there is an emphasis on the technological development of embedding sensor into wearable, such as shoes [29,30] and insole [31], to monitor gait performance for health monitoring in geriatric [32] patients and people with movement disorders [33,34,35,36]. However, few studies have highlighted the use gait parameters to assess frailty.

Assessment of gait parameter, such as walking speed, can give rise to accurate classification of frailty in older adults [16]. Previous studies have shown that tracking sensor-derived gait parameters, such as maximum swing velocity of the shank [27], gait speed [37,38], and gait variability [39] could be used for classification of non-frail, pre-frail, and frail older adults; yet, few have examined the role of the gait parameters during the propulsion phase of walking in frailty assessment. During this phase of walking, the body is generating the majority of the push-off force to propel the body forward. It is estimated that the force that is generated during the propulsion phase accounts for more than 85% of the metabolic cost during a gait cycle [40]. Therefore, assessment of the propulsion phase might provide clinically-relevant correlation with the stage of frailty in a person.

The deterioration of gait performance during the propulsion phase can embody the five frailty phenotypes that were proposed by Fried et al. [13]. For example, the weakness in propulsive muscle group is similar to grip force decline mentioned in the weakness phenotype [41]. The loss of muscles mass (due to atrophy or dystrophy) may be correlated to shrinking phenotype (i.e., unintentional weight loss). As the result of weakness and muscle loss, the push-off force during the propulsion phase may be reduced, and this could have resulted in a reduction in gait speed, which is indicative of slowness phenotype [39], as well as physical fatigue (exhaustion phenotype) [42]. In addition to muscle weakness, the loss of propulsion could also be attributed to the reduction in the range of motion (ROM) as a result of the decline in joint flexibility. Toosizadeh et al. [19] had demonstrated that the elbow flexibility was significantly correlated with walking time, weakness, exhaustion, and low activity in the upper extremity. However, in the lower extremity, the joint stiffness not only affects the propulsion through the changes in flexion and extension of the ankle joint [43], but it also contributes to changes in ankle eversion and inversion, as well as tibia pronation and supination. These pieces of evidence suggest that gait parameters during the propulsion phase may be reliable indicators of physical frailty. Therefore, we designed a study to examine the role of gait parameters during the stance, swing, and propulsion phase to predict physical frailty. We hypothesize that gait parameters during the propulsion phase could be strong indicators of frailty status in the at-risk population. In addition, an artificial intelligence (AI) model was used to determine the reliability of the model to predicting the frailty status using a single-sensor system.

## 2. Materials and Methods

### 2.1. Participants

This is a cohort observational study. Participants were enrolled from geriatric outpatient clinical visits or community dwelling older adult settings. Inclusion criteria included older adults with age ≥55 years without significant gait or balance disorders, which may limit their ability to walk 20 m without aid. Therefore, participants who were able to walk with an assistive device, such as cane, were also included in the study. Our cutoff age was less than 65 years since we did not exclude those with chronic medical condition (e.g., diabetes mellitus, peripheral arterial disease, HIV, etc.), in whom geriatric symptoms were reported at earlier age [44,45,46]. Exclusion criteria were sign of severe cognitive impairment, which was evaluated using Mini-Mental State Examination (MMSE) [47]. Participants with MMSE score ≤16 and those who were unable or unwilling to consent were excluded from the study. Participants who met the eligibility criteria signed the written consent form. This study was approved by the local institutional review boards (IRBs).

### 2.2. Frailty Phenotype Assessment

Frailty was assessed using the frailty phenotype assessment that was developed by Fried et al. [13] based on the five phenotypes. These phenotypes are: shrinking, weakness, exhaustion, slowness, and low activity. Shrinking is characterized by an unintentional weight loss of 4.54 kg (10 lbs.) or more in the past year. Weakness was measured using a digital hand dynamometer (Camry Scale Store, City Industry, CA, USA). Participants were stratified for the presence of weakness phenotype using the lowest quintile (20%), based on gender and body mass index (BMI). Self-report exhaustion was evaluated using questions adopted from the Center for Epidemiologic Studies Depression questionnaire (CES-D). A 4.57 m (15 ft) walking test was used to measure slowness. Slowness was quantified by stratifying the walking speed by the slowest quintile based on gender and height. Self-reported level of physical activities was measured using the Minnesota Leisure Time Activity questionnaire [48]. Similar to self-report exhaustion and weakness, participants were stratified based on the lowest quintile.

Participants were classified by three level of frailty (non-frail, pre-frail, and frail) based on number of phenotypes presence in the participants. Non-frail participants exhibited zero phenotype. Pre-frail participants exhibited 1 to 2 of the five phenotypes. Participants were classified as frail if they had three or more phenotypes.

### 2.3. Sensor-Based Gait Assessment

**Figure 1:**
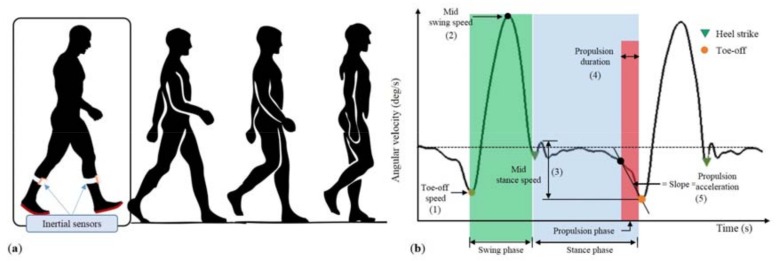
(**a**) Two wearable inertial sensors (LEGSysTM, BioSensics LLC) were attached to the left and right lower shin of the participant during a single walking task. The propulsion phase happens toward the end of the stance phase of the gait cycle. It is segmented between heel-off and toe-off. (**b**) Typical angular velocity profile of gait cycle in the sagittal plane derived from the inertial sensor during single walking task. The definition of each gait parameters are detailed in Table 1.

Participants were asked to perform one trial of a 4.57 m (15 ft) free and unobstructed single walking task (i.e., straight walking along a path) at their self-selected pace. Gait data during the single walking task were collected using the two LEGSys^TM^ inertial sensors (Biosensics LLC, Watertown, MA, USA) worn on the left and right lower shin (Figure 1a). Three-dimensional angular velocity data were collected at the sampling rate of 100 Hz wirelessly via Bluetooth. The x-axis of the sensor was aligned along the tibia. The angular velocity of the shin in the sagittal plane was calculated by using the *z*-component of the gyroscope (mediolateral axis). Typical angular velocity of the shin in the sagittal plane during a gait cycle is shown in Figure 1b. The gait cycle was segmented and extracted using the gyroscope data based on algorithms that were presented in previous studies [49,50,51,52]. Briefly, peak detection of the angular velocity in the sagittal plane (about the mediolateral axis) was used to identify the three phases of the gait cycle: swing phase, stance phase, and propulsion phase. Separate gait cycle data were extracted using the gyroscope data on the left and right lower shin. Participants with at least two gait cycles on each leg were included in the analysis. The average of each gait parameter during single walking task was calculated for each participants.

Based on suggested evidences, six gait parameters during the stance, swing, and propulsion phase were identified in the analysis to investigate their power to predict frailty status based on single walking task. These parameters are: toe-off speed, mid swing speed, mid stance speed, propulsion duration, propulsion acceleration, and speed norm. The visual description of these parameters during a gait cycle is shown in Figure 1b. The propulsion phase was defined as the initiation from heel-off to toe-off. This was segmented by detecting the steepest slope from toe-off to a stance position where heel-off begins. The toe-off speed was defined as the magnitude of the angular velocity at toe-off, while mid swing was the magnitude of the angular velocity at mid swing. The mid stance speed was defined as the magnitude of the largest difference in angular velocity during the stance phase (from heel-strike to toe-off). The propulsion duration was defined as the time difference from heel-off to toe-off. The angular acceleration was defined as the change in angular velocity (slope) during the propulsion phase. The speed norm was defined as the magnitude of the vector sum of the angular velocity in the frontal and transverse plane. The definition of these gait parameters is summarized in Table 1.

### 2.4. Neural Network Model

**Figure 2:**
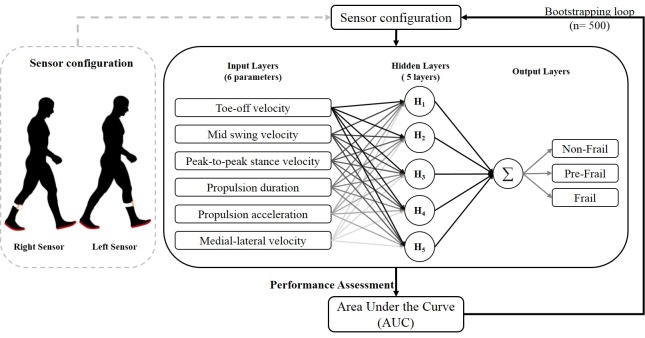
An eight-fold cross validation model with five hidden layers Artificial Neural Network was constructed to test the reliability and accuracy of classifying the frailty status of the participants in the study. Six selected gait parameters were identified and used as inputs to the model. The performance of the model was evaluated using the area under the curve. A 95% confidence interval was calculated to assess the reliability of the prediction.

A predictive model using a selected set of parameters was constructed using an Artificial Neural Network (ANN) algorithm (JMP^TM^, Cary, NC, USA) to assess its accuracy in predicting frailty status (Figure 2). This algorithm may help to evaluate whether a single-sensor system could be optimized to assess frailty in unsupervised setting. A *k*-fold cross validation study was designed where the participants were randomly separated into two groups: training data set and validation data set. The ANN network was constructed using 8-fold with 5 layers of hidden nodes. In the eight-fold scenario, participants were randomly divided into eight subsets (*n* = 20), where each subset was used as a validation set and the remaining participants (*n* = 141) was used as training data set. This process was repeated eight times using each subset as the validation data set. A hyperbolic tangent (TanH) activation function was used to represent the neuron that mapped the inputs to the outputs. Six selected gait parameters were used as the input layers to predict the frailty status of the validation data set after training. Participants were classified as either non-frail, pre-frail, or frail. The receiver of characteristic (ROC) curve [53,54] was generated for the validation set to assess the accuracy of the algorithm to correctly classify each frailty stage for the participants in the validation set. It was generated by plotting the true positive rate (sensitivity) against the false positive rate (1-specificity) for each frailty stage. The accuracy of the classification for each stage was measured by calculating the area under the ROC curve (AUC) [55]. The AUC represents the probability that a random participant from each group (non-frail, pre-frail, and frail) is correctly classified. An area of 1 represents a perfect classification of the frailty stage based on the algorithm and an area of 0.5 denotes a randomized classification. The robustness of the model was evaluated by calculating the 95% confidence interval using bootstrapping. With bootstrapping, a new training and validation set was randomly resampled from the population. The AUC for predicting frailty stages (non-frail, pre-frail, and frail) was recalculated for each resampled data set. The process was repeated 500 times. A 95% confidence interval of the classification of the non-frail, pre-frail, and frail was calculated using the bootstrapping results. The results from the AUC imply that we are 95% confident in predicting the stage of frailty of the participants by monitoring proposed gait parameters, such as propulsion duration and propulsion time during walking.

### 2.5. Statistical Analysis

Chi-squared test (χ^2^) was used to analyze the pairwise comparison of categorical variables, such as gender and fallers (faller vs. non-faller), in the non-frail, pre-frail, and frail group. The demographic, clinical characteristic, and gait parameters were analyzed using analysis of variance (ANOVA) and post hoc Game-Howell contrast was used for pairwise comparison assuming for unequal size and unequal variance. For comparison across three groups in ANOVA, the effect size eta squared (η^2^) was calculated [56]. The eta squared measures the proportion of variance in the dependent variables across different groups. Eta squared of 0.01 is considered to be small, 0.06 is medium, and 0.14 is large. These values are interpreted using percentage by multiplying the value by 100. For pairwise comparison, the effect size was calculated using Cohen’s effect size (d) [57]. Cohen’s effect size of 0.2 is considered to be small, 0.5 is medium, and 0.8 is large. The mean and the standard deviation were reported, unless otherwise noted. A univariate analysis of covariance (ANCOVA) was used to characterize the performance of these parameters among three groups, while adjusting for age, gender, and BMI. Linear regression model was used to analyze the relationship between the gait parameters and the frailty phenotypes, as proposed by Fried et al. [13]. The Spearman’s rho was used to calculate the correlation between the gait parameters and the frailty phenotypes [58]. For statistical analysis, the level of significance was set at alpha = 0.050.

## 3. Results

### 3.1. Demographic and Clinical Data

Using the inclusion criteria, 161 participants were selected for the study. Using Fried criteria, 30.4% (*n* = 49) of the participants were classified as non-frail, 57.2% (*n* = 92) was noted as pre-frail, and 12.4% (*n* = 20) were classified as frail [13] (Table 2). Chi-squared analysis showed that there were no significant differences between gender and fallers among the three groups. Age, which is often associated with frailty status, was matched among the non-frail (mean ± standard deviation, 71.2 ± 12.1 years old), pre-frail (74.6 ± 10.3 years old), and frail group (76.5 ± 14.3 years old). There were significant differences in weight between the non-frail versus pre-frail group (*p* = 0.030, d = 0.41) and pre-frail versus frail group (*p* = 0.003, d = 0.70); however, BMI was only significantly different between non-frail and pre-frail group (*p* = 0.030, d = 0.43). Pairwise comparison of cognitive performance showed significant differences in the MMSE score between frail versus non-frail (*p* = 0.009, d = 0.62) and frail versus pre-frail (*p* = 0.049, d = 0.46). Depression was significantly different in the frail group as compared to non-frail (*p* = 0.001, d = 1.17) and pre-frail (*p* = 0.001, d = 0.88). Non-frail participants had significantly less concern for fall when compared to the pre-frail (*p* = 0.001, d = 0.60) and frail group (*p* = 0.019, d = 1.72). Lastly, pre-frail individual exhibited more comorbidities than non-frail (*p* = 0.006, d = 1.27); however, there was no significant difference with frail individuals.

### 3.2. Sensor-Based Assessment of Frailty

The selected gait parameters (e.g., propulsion duration, propulsion acceleration, mid stance speed, speed norm, toe-off speed, and mid swing speed) were evaluated using data from the left and right sensor separately. The results are summarized in Table 3. ANOVA was used to analyze the discriminating power of the selected parameters to differentiate among non-frail, pre-frail, and frail group. The results showed that there were significant differences among the three groups for all of the selected parameters with *p* < 0.001 and effect size eta squared (η^2^) was between 0.09–0.24. This indicates that the selected gait parameters accounted for between 9.0–24.0% of the variance in the sample. Propulsion related parameters, such as propulsion duration, propulsion acceleration, and toe-off speed all demonstrated significant correlations among the three groups when using only the left or right sensor worn on the shin.

Gait performance data from the right sensor demonstrated that the frail group had longer propulsion duration when compared to the pre-frail (difference = +34%, *p* = 0.035, d = 1.01) and non-frail group (+57%, *p* = 0.003, d = 1.55). The propulsion acceleration was also significantly lower in the frail group as compared to the pre-frail (+54%, *p* = 0.002, d = 0.87) and non-frail (+84%, *p* < 0.001, d = 1.28). The toe-off speed was also significantly reduced in frail individuals when compared to pre-frail (+43%, *p* = 0.038, d = 0.65) and non-frail group (+66%, *p* = 0.00, d = 0.58). The mid swing speed was significantly lower in the frail group when compared to the non-frail (+46%, *p* < 0.001, d = 1.63) and pre-frail (+25%, *p* = 0.007, d = 0.84) group. The mid stance speed (non-frail vs. pre-frail) and speed norm (pre-frail vs. frail) yielded no statistical significance; however, there was a reduction in both velocities between groups, 11% and 24%, respectively. Using data from the sensor that was worn on the left shin, only the pairwise comparison of the propulsion duration between the frail and non-frail yielded no statistical significance (*p* = 0.071); however the effect size was large (d = 1.81).

**Figure 3:**
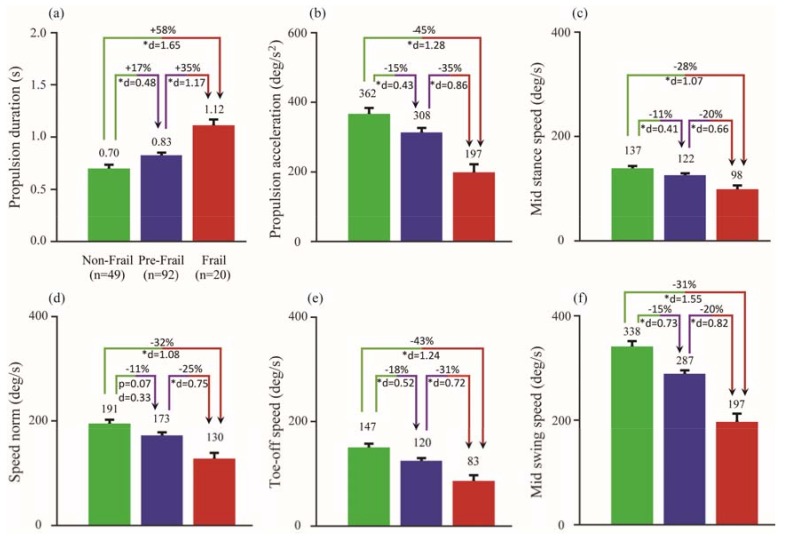
Result of univariate analysis of covariance for the six gait parameters (**a**–**f**) adjusting for gender, age, and BMI for the right sensor. * indicates statistical significance with alpha = 0.050. Percentage differences and Cohen’s effect size (d) were also calculated in each pairwise comparison.

The sensitivity of these gait parameters to age, gender, and BMI were investigated using a univariate analysis of covariance to account for these covariates. The results are shown in Figure 3. Age, sex, and BMI did not alter the effect on the performance of these gait parameters. Thus, the sensor-derived gait parameters remain significantly different among the three groups.

Correlation of the selected parameters with frailty phenotypes that were proposed by Fried et al. [13] were analyzed using Spearman’s correlation (*rho*). The correlation and the statistical results are summarized in Table 4. Using only the data from the right worn sensor, the results demonstrated that propulsion related parameters were highly correlated with several phenotypes. For example, propulsion duration was positively correlated with weakness (*rho* = 0.360, *p* < 0.001), slowness (*rho* = 0.684, *p* < 0.001), and exhaustion (*rho* = 0.237, *p* = 0.023). The propulsion acceleration was negatively correlated with weakness (*rho* = −0.257, *p* = 0.013) and slowness (*rho* = −0.645, *p* < 0.001). The mid stance speed was only correlated to slowness (*rho* = −0.553, *p* < 0.001); however, the speed norm was negatively correlated to weakness (*rho* = −0.330, *p* = 0.001), slowness (*rho* = −0.543, *p* < 0.001), exhaustion (*rho* = −0.248, *p* = 0.017), and low activity (*rho* = −0.212, *p* = 0.043). Speed norm was correlated to all frailty phenotype except shrinking. Toe-off speed was negatively correlated to weakness (*rho* = −0.402, *p* < 0.001), slowness (*rho* = −0.646, *p* < 0.001), and exhaustion (*rho* = −0.205, *p* = 0.050). Lastly, mid swing speed was negatively correlated to weakness (*rho* = −0.358, *p* < 0.001) and slowness (*rho* = −0.784, *p* < 0.001). Similar results were also observed when using data from the sensor that was worn on the left shin.

### 3.3. Neural Network Modeling

**Figure 4:**
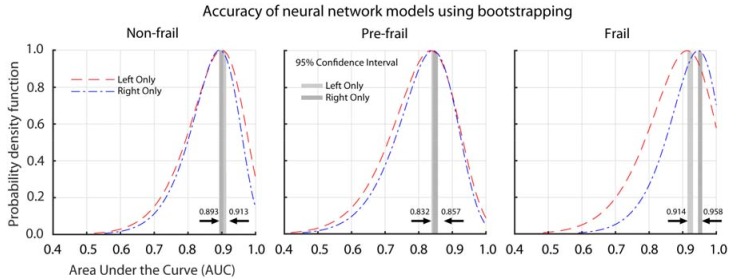
Probability distribution function the Area under the Curve (AUC) of two different sensor configurations. The confidence interval (shaded region) was calculated using bootstrapping (iteration = 500). The upper and lower limit of the confidence interval of the sensor configuration are also shown.

Bootstrapping (iteratio*n* = 500) was used on the neural network to find the 95% confidence interval of the frailty assessment using the data from the left and right shin sensor separately. Using data from the sensor worn on the left shin, the lower and upper bound of the AUC ranges from 0.900–0.913 for non-frail, 0.838–0.854 for pre-frail, and 0.914–0.931 for frail group. Using only gait data from the sensor worn on the right shin, the lower and upper bound limit of the AUC were 0.893–0.905 for non-frail, 0.842–0.857 for pre-frail, and 0.945–0.958 for frail group. The smallest AUC for the classification of frailty was in the pre-frail group when using gait data from either the left or right worn sensor (Figure 4).

## 4. Discussion

Assessment of frailty status is a critical component of delivering better healthcare to the aging population [59]. Timely treatment and intervention could be prescribed or recommended if frailty could be identified early. Currently, clinical assessment of frailty is performed in a supervised setting by trained personnel. Furthermore, current assessments are limited to classification such as non-frail, pre-frail, or frail. However, with the proliferation of wearable technology, embedding sensors into daily living activities and wearables devices could provide new avenues to obtain real-time assessment of frailty and greater granularity in the classification. In this study, we demonstrated the feasibility of using a foot-worn wearable sensor to monitor and detect different frailty stages in ambulatory adults using quantifiable gait characteristics with an emphasis on the propulsion phase of walking. The results could potentially be used to evaluate frailty status in unsupervised setting; allowing for future development of a single-sensor system to assess frailty assessment at home, clinic, and even outdoor environment.

Several wearable platforms have been developed to quantify physical frailty, to distinguish presence or absence of frailty phenotypes (e.g., slowness, exhaustion, weakness, low activity, and shrinking), and/or to differentiate different frailty stages (e.g., non-frail, pre-frail, and frail) [17,18,19,20,21,22,23,24,25,26,27,28]. These studies have mainly focused on quantifying frailty by measuring functional performance during different physical tasks, such as a 20s rapid elbow flexion-extension test [17,18,19], balance [20,21] and gait tests [22,23], physical activity monitoring [24,25], and postural transition test, such as the Timed Up and Go Test [26,27,28]. However, their proposed sensor location are not always suitable for remote and continuous monitoring of frailty outside of clinical setting or under unsupervised condition. Extracting frailty related parameters from sensors during lower extremity tasks could yield new opportunities for alternative form factors, such as different types of footwear (e.g., socks, shoes, braces, etc.), to monitor frailty during daily living.

Substantial works have been done to utilize gait performance to identify frailty [60,61,62]. For example, Castell et al. showed that people who are at higher risk of frailty had a lower walking speed [63] and that gait speed could be used as a predictor of adverse event outcome in older adults. In this study, we also observed a reduction in the mid swing speed in the non-frail (336.9 deg/s), pre-frail (−15%, 288.3 deg/s), and frail (−32%, 230.0 deg/s) participants. However, the focus on a single speed parameter might over simplify a multifaceted syndrome such as frailty. Schwenk et al. [16] suggested that parameters beyond gait speed could provide more granularity to the frailty assessment and adopted for different diseased population. For example, Thiede et al. [39] has demonstrated that frail individual with peripheral arterial disease tends to walk slower and in shorter steps. In this study, we observed a slower and weaker propulsive performance in those who were identified as frail when compared to pre-frail and non-frail group. For instance, the propulsion duration increased by an average of 17% as the non-frail participants become pre-frail, and it substantially increased by 58% in frail population, which could be a manifestation of slowness, weakness, and exhaustion phenotype. Furthermore, the propulsion acceleration dropped by 16% and 46% for pre-frail and frail group, when compared with non-frail. Propulsion acceleration was significantly correlated with weakness and slowness phenotype when using the sensor data from the right shin (Table 4). The inability to accelerate the body forward may be explained by advancing weakness in adults who become pre-frail or frail. This may be due to muscle loss from sarcopenia [23] (manifesting the shrinking phenotype) or fat infiltration and loss of muscle quality and force production capacity in older adults [64]. While monitoring for exhaustion was not directly possible as some participants had only a few completed gait cycles (two or more) in the 4.57 m (15 ft) walking test, the decline in angular velocity may indicate the progression of exhaustion since these movement are modulated by smaller lower body muscles (e.g., popliteus) that may become exhausted much faster and more frequent than the larger muscles (e.g., soleus). This hypothesis was observed in the correlation between speed norm and exhaustion phenotype (rho = −0.248, *p* = 0.017). Limitation of the movement of the speed norm has been shown to restrict the ability of the knee and ankle joint to generate maximum ground reaction force [65,66], which might be indicative of weakness and exhaustion frailty phenotype.

Frailty is a geriatric syndrome with high prevalence in older adults, and, as expected, the age of the non-frail participants was younger than the pre-frail and frail group (Table 1). However, there was no statistical difference in age among the three groups. The similarity in age for the three groups emphasizes the need for development of sensor-based algorithm for the continuous and standalone monitoring of older adults. Early detection of pre-frail status might create the possibility of reversing the condition or delaying the transition to frail status through intervention. This motivates the development of a deep learning algorithm [67,68] to assess frailty while using the propulsion phase parameters, which were found to be sensitive in identifying the three groups of non-frail, pre-frail, and frail across a diverse cohort (e.g., peripheral arterial disease, HIV, diabetes mellitus, etc.).

Two different sensor configurations were analyzed in this study. Data from the left and right sensor worn on the shin were evaluated separately. Using data collected from a single sensor (left or right), we were able to achieve accuracy, as defined using the AUC, between 84% (in pre-frail population) to 96% (in frail population). This indicates that the algorithm was able to classify frail participant at 96% accuracy using the gait parameters that were proposed in the study. These results suggested that proposed gait parameters are highly associated with frailty stages, especially in frail and non-frail participants (90% accuracy). As expected, the transition toward severe stages of frailty is more difficult to assess; however, our algorithm was able to classify pre-frail participants with 84% accuracy. The selected parameters are robust in predicting frailty, regardless of whether the sensor is attached to the left or the right shin. These results could potentially encourage the integration of a single-sensor system to assess frailty by measuring gait performance, which might be more suitable for unsupervised setting. Currently, clinical assessment [13,69] are limited to the classification of non-frail, pre-frail, and frail. However, for clinicians, the knowledge gaps in the gradation of the severity of frailty could dilute the effect of targeted intervention. More gradation of frail severity could provide complementary information to clinicians in making critical health care decisions [59,70,71]. Using deep learning algorithm can help to develop a more continuous scale, which might result in a more refined stratification of frailty status.

From sensor type point of view, we have demonstrated that all the parameters of interest could be extracted from a single gyroscope attached to lower extremities. This could make it more suitable for integration into smart footwear, such as shoes, socks, and braces. Unlike accelerometers, which are sensitive to sensor location [72,73] or pressure sensors that are sensitive to wear effect, and thus need regular calibration [74,75], gyroscope is insensitive to sensor-location as long as the segment is assumed to be rigid and it does not need regular calibration [50,73], thus making it more suitable for unsupervised setting. This also facilitates the deployment of the proposed algorithm in varieties of smart footwear, including smart sock (e.g., Sensoria smart socks, Sensoria Fitness, WA, USA), pressure offloading footwear (e.g., Optima Molliter, MC, Italy), ankle braces (e.g., Smart Moore Balance Brace, Orthotics Holdings Inc., AZ, USA), smart shoes (e.g., Sensoria Walk, Sensoria Fitness, WA, USA), and enhanced their capability to remotely monitor frailty status and its progression to different frailty stages without the need of frequent calibration or assessment by trained staff or under supervised condition. 

The method for deploying the proposed algorithm for the remote monitoring of frailty stages is beyond of the scope of this study. However, one potential deployment scenario of the algorithm could be to integrate a gyroscope sensor into a sock that is similar to the design that was proposed by Sensoria smart socks (Sensoria Fitness, WA, USA). Using a gyroscope, prolonged unbroken walking bouts (e.g., more than 20 consecutive steps) could be detected during the activity of daily living using the algorithm proposed by Aminian et al. [76]. Once these unbroken walking bouts are detected, the physical frailty could be quantified based on the propulsion performance using the model that is proposed in this study. The cutoff for 20 consecutive steps was selected based on a secondary analysis of the data reported by Moufawad el Achkar et al. [77]. Their study revealed that the average cadence estimated from walking bouts with more than 20 steps, measured during activities of daily living in non-frail older adults, was approximately 110 steps/minute and 90 steps/minute for walking bouts, with less than 20 consecutive steps. Schwenk et al. [22] analyzed gait data during a walking test that was performed at habitual speed and under supervised condition. Their study reported a cadence of 111 steps/minute for non-frail and 100 steps/minute for pre-frail individuals. Together, it could be speculated that daily unbroken walking bouts of greater than 20 consecutive steps might better represent the habitual walking speed than those with less than 20 consecutive steps. According to Najafi et al. [78,79], based on studies in people with diabetes, it is anticipated that over 50 bouts of walking per day with 20 steps or more could be collected in non-frail and pre-frail population. Thus, for determining progression in frailty stages (targeting non-frail and pre-frail population), the proposed scenario seems to be practical. But, this needs to be confirmed in another study. This scenario may also help to improve the accuracy by averaging the results of over 50 or more detected prolonged unbroken walking bouts during a day. To improve the autonomy and battery life, the system could be defaulted to be in sleep mode and only be activated when three consecutive steps are detected and return to sleep mode if walking is stopped before 20 steps or if it exceeds more than 30 steps. The cutoff for 30 steps was defined based on Lindemann et al. [80]. Their study suggested that after achieving steady state walking, which is often occurred before 10 consecutive steps, 20 consecutive steps and 40 consecutive steps resulted in the same average values for major gait parameters (e.g., stride length, stride time, etc.). Thus, it is speculated that walking longer than 30 consecutive steps may not influence the estimated propulsion performance. On the other hand, longer step count during prolonged walking bout may add a potential confounder that is associated with fatigue.

In this study, we analyzed over 161 participants; however, the population was more skewed toward the non-frail and pre-frail population. Even with a small sample size of frail participants, we were able to achieve significantly high effect size across the three groups. Similarly, we were limited to the number of gait cycle for each participant. A study by Lindemann et al. has suggested that steady-state walking in frail elderly does not occur until at least 2.5 m (8 ft) into walking, which is approximately six steps [80]. However, during a 4.57 m (15 ft) single task walking, the number of steady-state gait cycle was limited. Thus, the acceleration phase of the walking task might induce confounding factors during the propulsive phase of frail older adults. More importantly, these short gait cycles might not capture the exhaustion phenotype (fatigue) as suggested by Fried. Lastly, these data were collected under a structured single walking task and in a controlled laboratory environment, which might have an impact on gait performance. In a future study, we hope to apply the same model to gait data during unsupervised setting (using a single embedded sensor in footwear, offloading device, socks, etc.) to determine the efficacy of these parameters to assess frailty during daily living activities. Additionally, the engineering feasibility of integrating the proposed algorithm into wearable devices to assess frailty under unsupervised condition must be considered in future study; however it is beyond the scope of the current study. This study proposes a new algorithm to assess frailty using gait parameters with emphasis on the propulsion phase of the gait cycle. This development is an important facet in a multidisciplinary approach to develop smart footwear to monitor frailty.

## 5. Conclusions

This study demonstrates that a foot-worn sensor-derived gait measures during the propulsive phase of walking can be sensitive metrics in assessing frailty. Using these metrics, we have developed and validated a predictive model that could be used for unsupervised and real-time assessment using wearable sensor. These results could motivate the development and integration of single-sensor system into wearable footwear in order to assess frailty during daily living activities.

## Figures and Tables

**Table 1:** Definition of the sensor-derived gait parameters.

Sensor-Derived Gait Parameters	Unit	Description
Toe-off speed	degree/s	Magnitude of angular velocity at toe-off (Figure 1b, 1)
Mid swing speed	degree/s	Magnitude of angular velocity at mid swing (Figure 1b, 2)
Mid stance speed	degree/s	The magnitude of maximum range of the angular velocity during stance phase (Figure 1b,3)
Propulsion duration	second (s)	Duration of time from heel-off to toe-off in a gait cycle (Figure 1b, 4)
Propulsion acceleration	degree/s^2^	The average angular acceleration (slope) during the propulsion phase (Figure 1b, 5)
Speed norm	degree/s	The magnitude of the vector sum of the angular velocity in the transverse and frontal plane

The mean, standard deviation, and coefficient of variation were calculated for all of the sensor-derived gait parameters. Toe-off, mid swing, and mid stance speed denote rotation in the sagittal plane.

**Table 2:** Demographic and clinical characteristic of participants.

Characteristic	Non-Frail (N) (*n* = 49)	Pre-Frail (P) (*n* = 92)	Frail (F) (*n* = 20)	*p*-Value (*η^2^*)	Pairwise Comparison *p*-Value (d)		
					N-P	N-F	P-F
Gender ^+^							
Male *n* (%)	17 (34.7)	41 (44.6)	7 (35.0)		0.260	0.981	0.433
Female *n* (%)	32 (65.3)	51 (55.4)	13 (65.0)				
Age ^a^, years	71.2 (±12.1)	74.6 (±10.3)	76.5 (±14.3)	0.141 (0.025)	0.230 (0.31)	0.340 (0.41)	0.850 (0.17)
Height, m	1.66 (±0.09)	1.67 (±0.12)	1.60 (±0.12)	**0.023 (0.046)**	0.970 (0.04)	0.082 (0.66)	**0.052 (0.64)**
Weight, kg	73.5 (±15.5)	81.5 (±21.2)	67.6 (±14.3)	**0.003 (0.070)**	**0.030 (0.41)**	0.271 (0.31)	**0.002 (0.70)**
BMI, kg/m^2^	26.5 (±5.3)	29.2 (±6.5)	26.5 (±5.4)	**0.025 (0.046)**	**0.030 (0.43)**	0.990 (0.02)	0.131 (0.43)
History of fall ^+^ *n* (%)	14 (28.6)	38 (20.7)	7 (35.0)		0.973	0.392	0.394
Cognition performance (MMSE)	29.0 (±1.3)	28.5 (±1.7)	27.4 (±3.2)	**0.032 (0.069)**	0.278 (0.19)	**0.009 (0.62)**	**0.049 (0.46)**
Depression (CES-D)	7.0 (±7.0)	9.0 (±8.0)	16.6 (±6.8)	**0.001 (0.15)**	0.215 (0.17)	**0.001 (1.17)**	**0.001 (0.88)**
Concerns for falls (FES-I)	20.9 (±3.8)	28.8 (±11.9)	34.1 (±17.0)	**0.001 (0.15)**	**0.001 (0.60)**	**0.019 (1.72)**	0.486 (0.43)
# of comorbidity	2.0 (±1.7)	3.4 (±2.0)	4.8 (±1.9)	**0.002 (0.166)**	0.071 (0.39)	**0.006 (1.27)**	0.125 (0.65)

^+^ Gender and history of fall were evaluated using chi-squared (χ^2^). ^a^ The mean ± standard deviation is presented here, unless denoted otherwise. *n* represents the number of sample in each group. Post hoc Games-Howell test was used for pairwise comparison with alpha = 0.050. For three group comparisons, the effect size eta squared (η^2^) was calculated. Statistical significant interaction are highlighted with bold type.

**Table 3:** Sensor-derived gait parameters during single walking task across three groups.

Gait Parameters	Group	Mean ± Std	*p*-Value (η^2^)	Pairwise Comparison			
				Group	*p*-Value (*d*)	Mean Difference 95% CI	
***Right Sensor***						Lower	Upper
Propulsion duration (s)	Non-frail	0.70 ± 0.11	**<0.001 (0.20)**	N-P	<0.001 (0.62)	−0.19	−0.55
	Pre-frail	0.83 ± 0.23		N-F	0.003 (1.55)	−0.67	−0.14
	Frail	1.11 ± 0.46		P-F	0.036 (1.00)	−0.55	−0.17
Propulsion acceleration (deg/s^2^)	Non-frail	366.6 ± 137.5	**<0.001 (0.13)**	N-P	0.035 (0.46)	3.4	115.8
	Pre-frail	306.0 ± 125.4		N-F	<0.001 (1.28)	90.6	243.1
	Frail	198.7 ± 109.8		P-F	0.002 (0.87)	38.8	175.8
Mid stance speed (deg/s)	Non-frail	137.6 ± 42.2	**<0.001 (0.10)**	N-P	0.075 (0.42)	−1.2	32.2
	Pre-frail	122.1 ± 34.5		N-F	<0.001 (0.99)	16.6	62.2
	Frail	98.2 ± 32.3		P-F	0.016 (0.70)	4.0	43.8
speed norm (deg/s)	Non-frail	196.4 ± 53.5	**<0.001 (0.11)**	N-P	0.025 (0.46)	2.7	49.0
	Pre-frail	170.6 ± 57.7		N-F	0.003 (1.12)	22.2	112.1
	Frail	129.2 ± 3.6		P-F	0.067 (0.68)	−2.4	85.1
Toe-off speed (deg/s)	Non-frail	149.4 ± 48.2	**<0.001 (0.13)**	N-P	0.003 (0.58)	9.4	52.1
	Pre-frail	118.6 ± 55.3		N-F	<0.001 (1.32)	31.4	101.9
	Frail	82.7 ± 56.1		P-F	0.038 (0.65)	1.8	70.1
Mid swing speed (deg/s)	Non-frail	336.9 ± 63.3	**<0.001 (0.20)**	N-P	<0.001 (0.73)	21.2	76.0
	Pre-frail	288.3 ± 69.0		N-F	<0.001 (1.63)	61.9	151.9
	Frail	230.0 ± 74.8		P-F	0.007 (0.84)	15.0	101.7
***Left Sensor***							
Propulsion duration (s)	Non-frail	0.69 ± 0.10	**<0.001 (0.17)**	N-P	<0.001 (0.94)	−0.21	−0.65
	Pre-frail	0.83 ± 0.25		N-F	0.004 (4.02)	−0.63	−0.12
	Frail	1.07 ± 0.45		P-F	0.071 (1.83)	−0.50	0.19
Propulsion acceleration (deg/s^2^)	Non-frail	382.8 ± 115.3	**<0.001 (0.12)**	N-P	0.007 (0.60)	15.8	121.0
	Prefrail	314.4 ± 142.3		N-F	<0.001 (2.21)	82.2	242.9
	Frail	220.3 ± 126.5		P-F	0.016 (0.94)	15.4	172.8
Mid stance speed (deg/s)	Non-frail	144.1 ± 34.9	**<0.001 (0.10)**	N-P	0.037 (0.33)	0.8	31.9
	Pre-frail	127.8 ± 40.8		N-F	<0.001 (1.32)	18.6	64.9
	Frail	102.4 ± 35.8		P-F	0.023 (0.68)	3.1	47.8
Speed norm (deg/s)	Non-frail	198.8 ± 54.8	**<0.001 (0.12)**	N-P	0.041 (0.37)	0.8	48.6
	Pre-frail	174.1 ± 60.5		N-F	<0.001 (1.56)	36.4	101.9
	Frail	129.6 ± 48.9		P-F	0.004 (0.85)	13.4	75.4
Toe-off speed (deg/s)	Non-frail	154.2 ± 53.8	**0.001 (0.08)**	N-P	0.061 (0.34)	−0.8	47.6
	Pre-frail	130.8 ± 64.4		N-F	<0.001 (1.37)	25.9	93.8
	Frail	94.4 ± 51.8		P-F	0.027 (0.66)	3.6	69.3
Mid swing speed (deg/s)	Non-frail	347.6 ± 58.9	**<0.001 (0.24)**	N-P	<0.001 (0.78)	29.7	82.4
	Pre-frail	291.6 ± 69.2		N-F	<0.001 (2.59	72.4	176.3
	Frail	223.2 ± 85.8		P-F	0.007 (1.19)	17.3	119.4

N = Non-frail, P = Pre-frail, and F = Frail. Effect size among the three groups were calculated using eta squared (η^2^) for ANOVA analysis. For pairwise comparison, the Cohen’s effect size (d) was calculated. Statistical significances were evaluated using alpha = 0.050 and highlighted with bold font. The pairwise confidence interval of the mean difference (Mean Difference 95% CI) is also present here.

**Table 4:** Correlations between Fried phenotypes and sensor-derived gait parameters.

Gait Parameters	Shrinking		Weakness		Slowness		Exhaustion		Low Activity	
*Right Sensor*	*rho*	*p*-Value	*rho*	*p*-Value	*rho*	*p*-Value	*rho*	*p*-Value	*rho*	*p*-Value
Propulsion duration (*s*)	0.181	0.085	**0.360**	**<0.001**	**0.684**	**<0.001**	**0.237**	**0.023**	0.154	0.142
Propulsion acceleration (deg/s^2^)	−0.141	0.180	**−0.257**	**0.013**	**−0.645**	**<0.001**	−0.200	0.056	−0.168	0.109
Mid stance speed (deg/s)	−0.068	0.520	−0.183	0.081	**−0.589**	**<0.001**	−0.129	0.219	−0.091	0.390
Speed norm (deg/s)	0.061	0.561	**−0.330**	**0.001**	**−0.543**	**<0.001**	**−0.248**	**0.017**	**−0.212**	**0.043**
Toe-off speed (deg/s)	0.060	0.572	**−0.402**	**<0.001**	**−0.646**	**<0.001**	**−0.205**	**0.050**	−0.202	0.054
Mid swing speed (deg/s)	−0.119	0.257	**−0.358**	**<0.001**	**−0.784**	**<0.001**	−0.175	0.095	−0.127	0.227
***Left Sensor***										
Propulsion duration (*s*)	0.094	0.370	**0.386**	**<0.001**	**0.732**	**<0.001**	**0.241**	**0.021**	0.119	0.261
Propulsion acceleration (deg/s^2^)	−0.027	0.802	**−0.305**	**0.003**	**−0.676**	**<0.001**	−0.156	0.137	−0.119	0.258
Mid stance speed (deg/s)	−0.007	0.950	−0.196	0.061	**−0.568**	**<0.001**	−0.106	0.316	−0.104	0.323
Speed norm (deg/s)	−0.094	0.370	**−0.415**	**<0.001**	**−0.533**	**<0.001**	**−0.223**	**0.033**	**−0.231**	**0.027**
Toe-off speed (deg/s)	−0.033	0.754	**−0.407**	**<0.001**	**−0.567**	**<0.001**	**−0.224**	**0.032**	−0.173	0.099
Mid swing speed (deg/s)	−0.126	0.231	**−0.369**	**<0.001**	**−0.724**	**<0.001**	−0.176	0.093	−0.112	0.287

Linear correlations between the gait parameters and Fried frailty phenotypes were evaluated using Spearman’s coefficient (*rho*). Statistical significance was assessed with alpha = 0.050 and are bolded in the table. The left and right sensor showed similar correlations with Fried frailty phenotypes.
